# COVISHIELD vaccine-induced thyroiditis: a case report

**DOI:** 10.1186/s13256-023-04279-0

**Published:** 2023-12-15

**Authors:** J. Sachin, Ravindra Shukla, Abhishek Anil, Aswini Saravanan, Sanjay Santhyavu, Shoban Babu Varthya, Sneha Ambwani, Surjit Singh

**Affiliations:** 1 Department of Pharmacology, All India Institute of Medical Sciences, Jodhpur, Rajasthan 342005 India; 2 Department of Endocrinology and Metabolism, All India Institute of Medical Sciences, Jodhpur, Rajasthan 342005 India; 3 Department of Nuclear Medicine, All India Institute of Medical Sciences, Jodhpur, Rajasthan 342005 India

**Keywords:** COVISHIELD, COVID-19 vaccine, Thyroiditis

## Abstract

**Background:**

The rapid development of coronavirus disease 2019 vaccines during the pandemic has left their long-term effects largely unknown. Instances of autoimmune and subacute thyroiditis showing features of autoimmune/inflammatory syndrome induced by adjuvants have been reported post-vaccination. This case report aims to highlight the autoimmune/inflammatory syndrome induced by adjuvants syndrome after coronavirus disease 2019 vaccination, drawing attention to a possible connection with thyroid dysfunction and urging for further thorough research.

**Case presentation:**

We present a case of thyroiditis induced by the COVISHIELD vaccine in a 37-year-old Indian woman. An apparently normal and healthy adult woman developed neck pain and easy fatigability 2 weeks after the second dose of COVISHIELD, which gradually increased and was associated with irritability, decreased sleep, excessive sweating, tremor, palpitation, and weight loss. She presented to the outpatient department after 1 week of symptoms and was evaluated with laboratory tests and imaging. She was diagnosed with thyroiditis due to the coronavirus disease 2019 vaccine and was treated with propranolol.

**Conclusion:**

This case report adds to the growing evidence of coronavirus disease 2019 vaccine-related thyroid issues. The development of thyroiditis is rare and underreported post-coronavirus disease 2019 vaccination; hence, research to evaluate the association of coronavirus disease 2019 vaccines with thyroid dysfunction needs to be done in the future.

**Supplementary Information:**

The online version contains supplementary material available at 10.1186/s13256-023-04279-0.

## Background

The novel coronavirus disease 2019 (COVID-19), which is caused by the virus severe acute respiratory syndrome coronavirus 2 (SARS-CoV-2), has resulted in pandemic worldwide. It created a devastating effect on the demographics of the world, causing 3.8 million deaths worldwide [1]. Multiple vaccines, each producing effects through different mechanisms, were developed rapidly, and at present, more than 1 billion people have been vaccinated. COVISHIELD is a monovalent vaccine developed by the Serum Institute of India. It consists of a single recombinant, replication-deficient chimpanzee adenovirus Oxford 1 (ChAdOx1) vector, which carries the genetic code for the S-glycoprotein of the coronavirus. Following its administration, the S-glycoprotein triggers localized activity, provoking the production of neutralizing antibodies and initiating a cellular immune response [2].

Several adjuvants are employed to enhance the vaccine responsiveness, and they can induce autoimmune and inflammatory responses by promoting immunogenic cross-reactivity in genetically predisposed individuals [3, 4]. Several autoimmune and subacute cases of thyroiditis have been documented after vaccine exposure, displaying characteristics akin to the autoimmune/inflammatory syndrome induced by adjuvants (ASIA) [3]. Very few cases of ASIA following COVID-19 vaccine have been reported [5, 6]. The endocrine system is primarily affected by COVID-19, with thyroid dysfunction being the most common endocrine condition following vaccination [7]. The aim of this case report is to bring attention to the potential connection between the COVID-19 vaccine and the emergence of thyroid dysfunction, thereby supplementing the existing occurrences and strengthening the evidential basis and consequently promoting more in-depth research into this subject.

## Case presentation

**Fig. 1:**
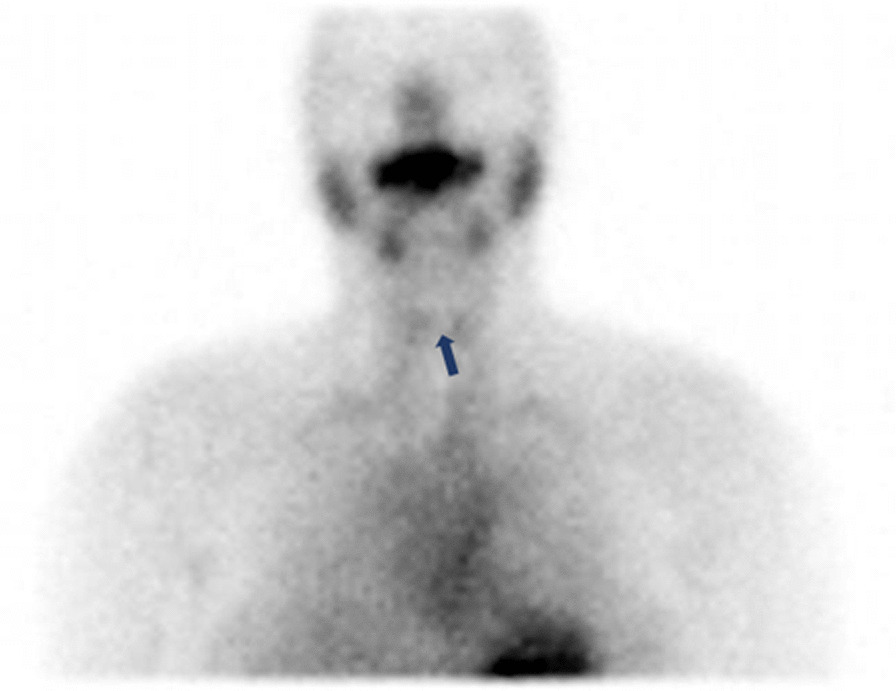
Thyroid scan showing thyroiditis. The arrow shows decreased uptake of the radio indicator (Technetium) within the thyroid gland, suggestive of thyroiditis

A 37-year-old Indian woman who was apparently normal before the COVID-19 vaccination presented to the outpatient department (OPD) with complaints of pain in the neck for 2 weeks, which were present throughout the day. This was accompanied by easy fatigability, irritability, decreased sleep, increased sweating, tremor, palpitation, and weight loss of approximately 3 kg in the previous 3 weeks. One week before the onset of these symptoms, she had been administered the second dose of the COVISHIELD vaccine. There were no records of any viral or respiratory sickness prior to the emergence of the symptoms. The patient received the first dose of COVISHIELD 3 months before the second dose. The patient had no history of adverse events to the first dose of COVISHIELD, except for 2 days of mild fever that resolved spontaneously. The patient had no history of any comorbidities such as diabetes mellitus, hypertension, or cardiac conditions. There was no personal or family history of autoimmune disorders. She was a non-smoker, non-alcoholic, and a teacher by profession. On thyroid examination, there was no visible swelling; however, upon palpation, tenderness was present. Primary hyperthyroidism was found in the thyroid function test. On ultrasound of the neck, multiple colloid cysts on the right lobe of the thyroid along with ill-defined hypoechoic areas in both the lobes were identified. Thyroid scan report was suggestive of thyroiditis with decreased uptake of technetium (Fig. 1). The results of the complete laboratory investigations performed for this patient during the first OPD visit have been provided in Table 1. The patient was diagnosed with thyroiditis induced by COVISHIELD and was treated with a beta blocker (propranolol) for 3 weeks to control palpitation and tremors. This incident was reported to the Indian Pharmacopoeia Commission (IPC) with a worldwide unique identification, IN-IPC-300617048. The patient adhered to the treatment and tolerated the drug well. She developed no side effects during the treatment period. All the presented symptoms resolved within a month, and her thyroid function test became normal. The patient developed no symptoms during the next 2 months of follow-up.

**Table 1:** Laboratory findings during initial OPD visit

Test	Result	Reference range
T3 (ng/mL)	2.42	0.58–1.59
T4 (micro g/dL)	19.46	4.82–15.65
TSH (mIU/L)	0.0050	0.34–5.60
Hemoglobin (g/dL)	12.5	12–16
RBC count (million/mm^3^)	4.14	3.8–4.8
Hematocrit (%)	36.4	36–48
MCV (fL)	88	83–101
MCH (pg)	30.3	26.4–33.2
MCHC (g/dL)	34.4	31.8–35.9
RDW CV (%)	12.4	11.6–14
WBC count (per mm^3^)	10,370	4000–10,000
Platelet count (per mm^3^)	336,000	150,000–410,000
MPV (fL)	8.8	7.5–10.3
Neutrophils (%)	75	40–80
Lymphocytes (%)	20	20–40
Eosinophils (%)	2	1–6
Monocytes (%)	3	2–10
Basophils (%)	0	0–2
Absolute neutrophil count (per mm^3^)	7778	2000–6700
ESR (mm/1 h)	25	0–21
Random blood sugar (mg/dL)	92	70–140
Anti-TPO (IU/mL)	3.175	1–16

*T3* triiodothyronine, *T4* thyroxine, *TSH* thyroid stimulating hormone, *RBC* red blood cell count, *MCV* mean corpuscular volume, *MCH * mean corpuscular hemoglobin, *MCHC* mean corpuscular hemoglobin concentration, *RDW* red cell distribution width, *WBC* white blood cell count, *MPV* mean platelet volume, *ESR* erythrocyte sedimentation rate, *TPO* thyroid peroxidase

## Discussion

The presented case underscores a notable clinical association between COVID-19 vaccination and the subsequent development of thyroiditis. This 37-year-old woman exhibited a constellation of symptoms within weeks of receiving the second dose of the COVISHIELD vaccine. The temporal proximity of symptom onset to vaccination, coupled with the absence of any preexisting comorbidities, suggests a plausible link between the vaccine and the thyroid dysfunction. Thyroid function tests indicated primary hyperthyroidism, further supporting the diagnosis of vaccine-induced thyroiditis. The ultrasound findings, along with thyroid scan, added weight to the diagnosis. Notably, the patient’s symptoms ameliorated within a month, indicating the self-limiting nature of the condition. This case contributes to the growing evidence regarding potential autoimmune responses triggered by COVID-19 vaccines, particularly involving thyroid-related sequelae.

While COVID-19 infection is known to be associated with thyroiditis [8, 9], COVID-19 vaccines were also found to cause subacute thyroiditis. Few subacute thyroiditis cases have been documented following the Pfizer-BioNTech messenger RNA (mRNA) vaccine administration [10], as well as with CoronaVac, which is composed of inactivated SARS-CoV-2 virus [5]. There were a few new cases of Graves’ disease after the administration of the Pfizer vaccine. These cases were thought to potentially result from an autoimmune inflammatory syndrome triggered by adjuvants [11]. Adjuvants play a crucial role within the vaccine composition that basically improves the response of the vaccine [12]. Nonetheless, a portion of individuals with a genetic predisposition may experience significant adverse reactions when adjuvants are introduced. This is attributed to the initiation of autoimmune responses and pathways, which disrupt the delicate immunological equilibrium within the host. Such disruption promotes the widespread activation of B lymphocytes through mechanisms such as molecular mimicry or other analogous etiopathogenic pathways [3].

While ASIA elucidates the underlying mechanism of thyroiditis due to adjuvants, AstraZeneca’s COVISHIELD, which is manufactured using genetically modified human embryonic kidney 293 cells [13], encompasses additional pathways contributing to vaccine-triggered thyroid inflammation. Notably, the angiotensin-converting enzyme 2 (ACE2) receptor has been identified as the point of entry for the SARS-CoV-2 spike protein into thyroid cells [14]. This might be the cause of immunization-mediated damage to the thyroid gland. Spike proteins of COVID-19 attach to ACE2 receptors present on the endothelium, resulting in inflammation of endothelial cells. This will cause downregulation of ACE2 receptors leading to decreased production of nitric oxide and secondary mitochondrial damage [15]. It is also known that the antibodies developed against the coronavirus have the potential to interact with antigens present in cells, including those in the thyroid [16]. Furthermore, spike proteins demonstrate molecular mimicry with thyroid peroxidase. Consequently, the antibodies prompted by COVID-19 vaccination engage with the surface receptors of the thyroid gland, leading to a transient thyroiditis that gradually resolves.

The strength of this case report lies in its documentation of the patient’s clinical presentation, laboratory findings, and response to treatment. The detailed thyroid function tests, ultrasound, and thyroid scan results provide comprehensive evidence to support the diagnosis. The reporting of this case to IPC adds value to the report, facilitating traceability and further investigation if necessary. Our case report was compiled in accordance with the Consensus-based Clinical Case Reporting (CARE) guidelines (Additional file 1). However, there are limitations to consider. This is a single case report, and thus causality cannot be definitively established. Individual variations in immune responses and underlying susceptibilities could contribute to the development of thyroiditis. Additionally, the long-term consequences or potential recurrence of thyroid dysfunction following subsequent vaccinations are yet to be determined. Despite these limitations, this case report raises an important flag for clinicians and researchers to monitor and investigate potential thyroid-related adverse events following COVID-19 vaccination.

## Conclusion

This case adds to the growing evidence of COVID-19-vaccine-related thyroid issues. While this report highlights the need for further investigation, it also serves as a reminder of the importance of continuous monitoring and prompt reporting of potential vaccine-related adverse effects.

### Supplementary Information

**Additional file 1.** CARE Checklist.

## Data Availability

Data sharing is not applicable to this article as no datasets were generated or analyzed during the current study. All necessary information related to the case report have been provided in the article. Waiver from the ethics committee and the patient’s consent form will be provided on reasonable request of the corresponding author.

## Abbreviations

COVID-19: Novel coronavirus disease 2019
SARS-CoV-2: Severe acute respiratory syndrome coronavirus 2
ChAdOx1: Chimpanzee adenovirus Oxford 1
ASIA: Autoimmune/inflammatory syndrome induced by adjuvants
OPD: Outpatient department
IPC: Indian Pharmacopoeia Commission
T3: Triiodothyronine
T4: Thyroxine
TSH: Thyroid stimulating hormone
ACE2: Angiotensin converting enzyme 2
