# Supplementary material for: COVISHIELD vaccine-induced thyroiditis: a case report
Source: J Med Case Rep. 2023 Dec 15;17:542. doi: 10.1186/s13256-023-04279-0 (PMC10722757; doi:10.1186/s13256-023-04279-0)
Supplement: Supplementary file 1 — Additional file 1. CARE Checklist. [file 13256_2023_4279_MOESM1_ESM.pdf]

CARE
case report guidelines

## CARE Checklist of information to include when writing a case report

(cc) BY-NC-ND

✓

| Topic | Item | Checklist item description | Reported on Line |
|---|---|---|---|
| **Title** | **1** | The diagnosis or intervention of primary focus followed by the words "case report" | ✓ 1 |
| **Key Words** | **2** | 2 to 5 key words that identify diagnoses or interventions in this case report, including "case report" | ✓ 3 |
| **Abstract (no references)** | **3a** | Introduction: What is unique about this case and what does it add to the scientific literature? | ✓ 3 |
| | **3b** | Main symptoms and/or important clinical findings | ✓ 3 |
| | **3c** | The main diagnoses, therapeutic interventions, and outcomes | ✓ 3 |
| | **3d** | Conclusion—What is the main "take-away" lesson(s) from this case? | ✓ 3 |
| **Introduction** | **4** | One or two paragraphs summarizing why this case is unique (**may include references**) | ✓ 4 |
| **Patient Information** | **5a** | De-identified patient specific information. | ✓ 5 |
| | **5b** | Primary concerns and symptoms of the patient. | ✓ 5 |
| | **5c** | Medical, family, and psycho-social history including relevant genetic information | ✓ 5 |
| | **5d** | Relevant past interventions with outcomes | |
| **Clinical Findings** | **6** | Describe significant physical examination (PE) and important clinical findings. | ✓ 5 |
| **Timeline** | **7** | Historical and current information from this episode of care organized as a timeline | ✓ 5 |
| **Diagnostic Assessment** | **8a** | Diagnostic testing (such as PE, laboratory testing, imaging, surveys). | ✓ 5,6 |
| | **8b** | Diagnostic challenges (such as access to testing, financial, or cultural) | |
| | **8c** | Diagnosis (including other diagnoses considered) | ✓ 5 |
| | **8d** | Prognosis (such as staging in oncology) where applicable | ✓ 5 |
| **Therapeutic Intervention** | **9a** | Types of therapeutic intervention (such as pharmacologic, surgical, preventive, self-care) | ✓ 5 |
| | **9b** | Administration of therapeutic intervention (such as dosage, strength, duration) | ✓ 5 |
| | **9c** | Changes in therapeutic intervention (with rationale) | |
| **Follow-up and Outcomes** | **10a** | Clinician and patient-assessed outcomes (if available) | ✓ 5 |
| | **10b** | Important follow-up diagnostic and other test results | |
| | **10c** | Intervention adherence and tolerability (How was this assessed?) | ✓ 5 |
| | **10d** | Adverse and unanticipated events | ✓ 5 |
| **Discussion** | **11a** | A scientific discussion of the strengths AND limitations associated with this case report | ✓ 9 |
| | **11b** | Discussion of the relevant medical literature **with references**. | ✓ 8,9 |
| | **11c** | The scientific rationale for any conclusions (including assessment of possible causes) | ✓ 10 |
| | **11d** | The primary "take-away" lessons of this case report (without references) in a one paragraph conclusion | ✓ 10 |
| **Patient Perspective** | **12** | The patient should share their perspective in one to two paragraphs on the treatment(s) they received | |
| **Informed Consent** | **13** | Did the patient give informed consent? Please provide if requested | **Yes** ☑ **No** ☐ |
